# Developing an ancient epithelial appendage: FGF signalling regulates early tail denticle formation in sharks

**DOI:** 10.1186/s13227-017-0071-0

**Published:** 2017-05-02

**Authors:** Rory L. Cooper, Kyle J. Martin, Liam J. Rasch, Gareth J. Fraser

**Affiliations:** Department of Animal and Plant Sciences, and the Bateson Centre, University of Sheffield, Sheffield, S10 2TN UK

**Keywords:** Homology, Shark, Epithelial appendage, Anatomical placode, Dermal denticle

## Abstract

**Background:**

Vertebrate epithelial appendages constitute a diverse group of organs that includes integumentary structures such as reptilian scales, avian feathers and mammalian hair. Recent studies have provided new evidence for the homology of integumentary organ development throughout amniotes, despite their disparate final morphologies. These structures develop from conserved molecular signalling centres, known as epithelial placodes. It is not yet certain whether this homology extends beyond the integumentary organs of amniotes, as there is a lack of knowledge regarding their development in basal vertebrates. As the ancient sister lineage of bony vertebrates, extant chondrichthyans are well suited to testing the phylogenetic depth of this homology. Elasmobranchs (sharks, skates and rays) possess hard, mineralised epithelial appendages called odontodes, which include teeth and dermal denticles (placoid scales). Odontodes constitute some of the oldest known vertebrate integumentary appendages, predating the origin of gnathostomes. Here, we used an emerging model shark (*Scyliorhinus canicula*) to test the hypothesis that denticles are homologous to other placode-derived amniote integumentary organs. To examine the conservation of putative gene regulatory network (GRN) member function, we undertook small molecule inhibition of fibroblast growth factor (FGF) signalling during caudal denticle formation.

**Results:**

We show that during early caudal denticle morphogenesis, the shark expresses homologues of conserved developmental gene families, known to comprise a core GRN for early placode morphogenesis in amniotes. This includes conserved expression of FGFs, sonic hedgehog (*shh*) and bone morphogenetic protein 4 (*bmp4*). Additionally, we reveal that denticle placodes possess columnar epithelial cells with a reduced rate of proliferation, a conserved characteristic of amniote skin appendage development. Small molecule inhibition of FGF signalling revealed placode development is FGF dependent, and inhibiting FGF activity resulted in downregulation of *shh* and *bmp4* expression, consistent with the expectation from comparison to the amniote integumentary appendage GRN.

**Conclusion:**

Overall, these findings suggest the core GRN for building vertebrate integumentary epithelial appendages has been highly conserved over 450 million years. This provides evidence for the continuous, historical homology of epithelial appendage placodes throughout jawed vertebrates, from sharks to mammals. Epithelial placodes constitute the shared foundation upon which diverse vertebrate integumentary organs have evolved.

**Electronic supplementary material:**

The online version of this article (doi:10.1186/s13227-017-0071-0) contains supplementary material, which is available to authorized users.

## Background

The diversity of phenotypes among vertebrate epithelial appendages is vast and includes disparate structures of the integument such as feathers, hair, scales and teeth [1, 2]. These organs have evolved to facilitate wide-ranging aspects of survival and reproduction. Despite such diversity, these structures generally develop from patterns of reciprocal interactions between two adjacent tissue layers: the epithelium and underlying mesenchyme [1]. Where scale-like structures also arise from more derived mechanisms, for example the physical cracking of highly keratinised crocodile skin to form randomly spaced, polygonal head scales, placode-derived scales are also present on the body [3].

Recent research has revealed shared ancestry among amniote epithelial appendages, based on the observation that reptilian scales, avian feathers and mammalian hair share a common foundation during early development [4]: the anatomical placode. This structure is characterised by conserved molecular markers and columnar epithelial cells with a reduced rate of proliferation. Placodes constitute a localised thickening of the epithelium together with an underlying dermal condensate (mesenchyme) [5, 6]. Morphogenesis of the placode results in the adult form [7] and is controlled by molecular signals that participate in a complex gene regulatory network (GRN). This placode GRN is thought to be largely conserved throughout amniotes [4, 8]. However, there is a gap in our knowledge regarding the developmental processes guiding placode morphogenesis in non-amniote vertebrates. It is not known whether this GRN is conserved across all jawed vertebrates [9].

Chondrichthyans (sharks, rays and chimaeras) are the sister lineage of osteichthyans and occupy a basal position in jawed vertebrate phylogeny. They possess hard, mineralised epithelial appendages known as odontodes. Odontodes include both teeth and dermal denticles and have been observed in early vertebrates that lived as long as 450 million years ago [10, 11]. Odontodes consist of a central pulp cavity surrounded by a dentine layer, encased within an enameloid (enamel-like) covering [12, 13]. Recent work has provided new genetic evidence for the old hypothesis that teeth and denticles share deep homology and that their development is controlled by a common odontode GRN [14]. Since their likely origin as a form of body armour [15], denticles have evolved to fulfil a plethora of functions: they reduce abrasive damage [16], aid feeding [17], deter parasites [18], enable communication [19] and improve hydrodynamic efficiency [20–22]. Chondrichthyan denticles exhibit broad morphological variation to facilitate these roles [13].

**Fig. 1:**
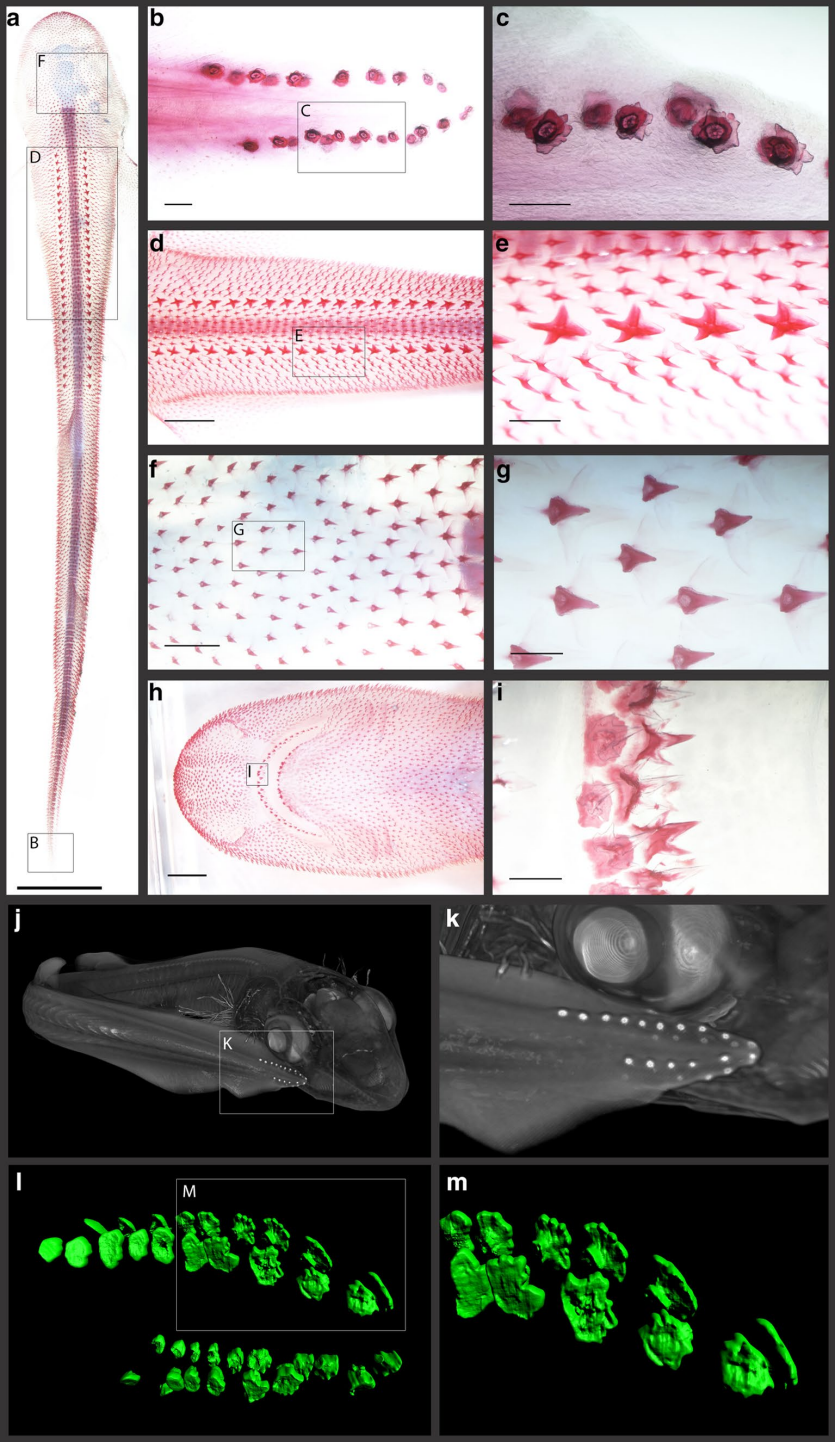
Odontode diversity of the pre-hatchling Catshark (*S. canicula*). Samples *a*–*i* are cleared and stained for calcium-rich tissue using alizarin red dye. Samples *j*–*k* are computerised tomography (CT) scans of a Stage 32 whole embryo, and samples *l*–*m* are light sheet fluorescence microscopy (LSFM) images of caudal denticles of a Stage 31 embryo, stained with alizarin red. The pre-hatchling (*a*) possesses three major external denticle types. The caudal denticles are the first to emerge, appearing on either side of the tip of the tail in dorsal and ventral rows (*b*, *c*, *j*–*m*) [23]. These denticles are not strongly polarised, although cusps generally point towards the posterior [27]. Next, the dorsal denticles emerge along the trunk of the embryo in two polarised rows (*d*, *e*). Finally, general body denticles emerge just before hatching, covering the whole body (*f*, *g*). These denticles are also highly polarised. Teeth emerge in the jaws at a similar stage to general body denticles (*h*, *i*). The *scale bar* for *a* = 1000 µm, *b*, *c*, *g* and *i* = 200 µm, *d* and *h* = 2500 µm, *e* and *f* = 500 µm

In the *S. canicula* embryo, this variation can broadly be categorised into 3 classes: (1) the precocious embryonic denticles of the caudal tail, (2) the dorsal trunk and (3) adult type general body denticles (Fig. 1) [14]. Dorsal denticles (Fig. 1d, e) appear in two polarised rows at approximately 60–80 days post-fertilisation (dpf; Stage 31) and may trigger the subsequent emergence of general body denticles [23], as observed during feather tract patterning [24]. They are subsumed into general scalation soon after hatching [14]. General body denticles (Fig. 1f, g) are the most prevalent denticle type, appearing just before hatching at 145–175 dpf (Stage 34) [23], covering the skin in an intricate pattern when space is available and not in discrete rows [25, 26]. Before dorsal and body denticles appear, four rows of caudal denticles emerge at 52–60 dpf (Stage 30) [23]; two rows are present (dorsal and ventral) laterally on either side of the tail fin tip (Fig. 1b, c, j–m) [27]. Caudal denticle number can vary between 9 and 13 units which form on either dorsal row, and between 5 and 10 units which form on either ventral row [23]. The placodes of these denticles consist of a squamous epithelium overlying a basal epithelial layer of columnar cells, with condensing underlying mesenchyme (Fig. 3). They develop sequentially from posterior to anterior, approximately equidistant from one another [23, 27–29]. During morphogenesis, these denticles also mineralise in a posterior to anterior progression [23]. Despite being patterned in rows similarly to dorsal denticles, they display an irregular petaliform shape with variation in cusp number and have a less restricted polarity than other denticle types (Fig. 1l, m). These units have a dentine collar fusing the main cusp to the simple base [27], anchored within the mesenchymal dermis via connective tissues. Caudal denticles are transient epithelial structures that are lost before or during the hatching phase when general body denticles develop to take over their positions. This morphological disparity between caudal denticles and other denticle types from the dorsal trunk and general body extends beyond their macrostructure.

Caudal denticles contain a dentine type that shares histological similarity to dentine from odontodes of the Ordovician and Silurian Periods [10, 11, 27]. Unlike the orthodentine observed in the general body denticles of *S. canicula*, the tubules of this dentine exhibit a branching pattern [27, 30]. The combination of this primitive-type dentine composition and the regulated, iterative patterning mechanism of these denticles, which are found in sharks at least across families Scyliorhindae and Heterodontidae, indicates that caudal denticles may have a deep phylogenetic history and have been retained in extant sharks over 450 million years of evolution [27, 28]. However, little is known about the developmental processes or GRN underlying the formation of caudal denticles or indeed other denticle types in chondrichthyans.

Fibroblast growth factor (FGF) signalling is essential for various aspects of both embryogenesis and adult homeostasis, such as tissue repair and regeneration [31]. FGFs have essential roles throughout vertebrate organogenesis, for example in limb, lung and brain development [32–34]. They mediate their responses by activating cell surface tyrosine kinase FGF receptors (FGFRs) [31]. FGF signalling is also widely involved in the development of taxonomically diverse epithelial appendages of the integumentary system, such as hair, feathers, scutes, scales and teeth [35–39]. Relative to the epithelial appendages of amniotes, little is known about the GRN controlling shark denticle placode formation, although some recent work has documented signalling during shark tooth development [40] and compared it to development of other odontode types [14, 41, 42].

During feather placode development, ligands of the FGF signalling family (such as Fgf4) work together with sonic hedgehog (Shh) in a positive feedback loop, that promotes expression of both *Fgf4* and *Shh* whilst also inducing expression of bone morphogenetic protein 4 (*Bmp4*) [36, 43]. *Bmp4* then has an inhibitory effect upon both *Shh* and *Fgf4*, downregulating their expression to control patterning by limiting placode formation exclusively to the site of future organs [36, 44, 45]. This inhibitory action of mesenchymal *Bmp4* has also been observed during mouse hair development [46]. The mesenchymal expression of *Bmp4* is conserved during morphogenesis throughout amniote epithelial appendage development [4]. It is unknown whether this FGF, Shh and Bmp4 signalling feedback system is conserved throughout all vertebrate epithelial appendage placode GRNs, although conservation of these markers is widely observed during amniote placode formation [4].

This study examines whether the molecular signalling observed during early morphogenesis of amniote integumentary organs is conserved within the development of caudal denticles of the shark (*S. canicula*). By comparing gene expression to the development of other epithelial appendages and using functional experiments to examine gene interactions, it is possible to infer putative GRN relationships [14]. A combination of anatomical, histological and molecular techniques including whole mount in situ hybridisation and immunohistochemistry was used to examine the development of shark caudal denticles, focusing on the role of the FGF signalling pathway and associated members of the putative core conserved placode GRN, inferred from studies in amniotes. To study the conservation of placode GRN members between different odontode types, gene expression was also examined during development of general body denticles. The fibroblast growth factor receptor (FGFR) inhibitor SU5402 was used to examine the effect of suppressing signalling of this major developmental pathway. By examining the role of FGF signalling during epithelial appendage development in a chondrichthyan model and its effects upon the expression of other putative GRN members, it will be possible to elucidate the degree to which epithelial integumentary organ GRNs are conserved across jawed vertebrates, and evaluate their potential homology.

## Results

### Caudal denticle placode development reveals conserved morphogenetic mechanisms for integumentary organ formation

**Fig. 2:**
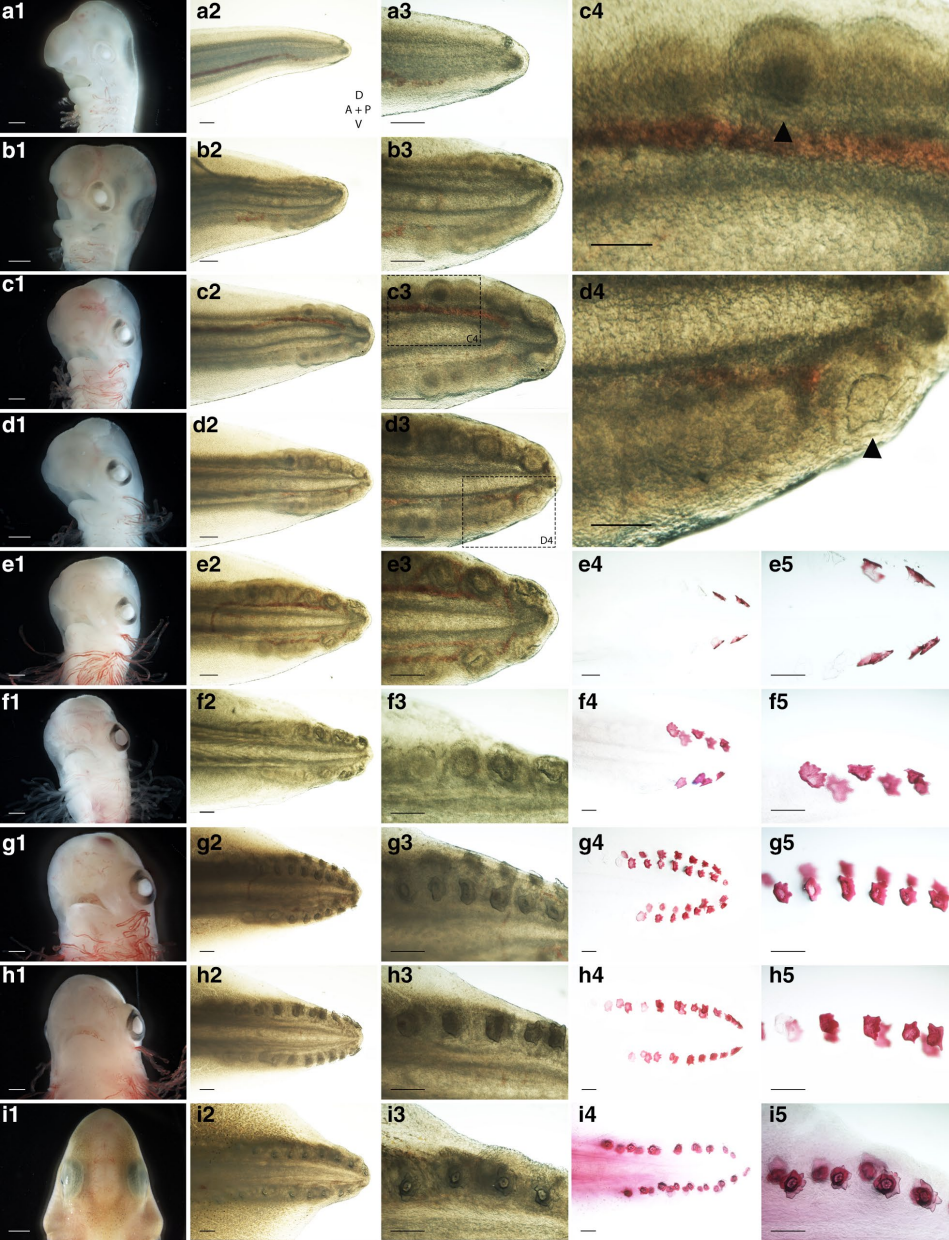
Sequential development of caudal denticles in the Catshark. As the embryo develops from Stage 27 (*a*) to Stage 33 (*i*), the gills proliferate, the eyes are encircled with pigment of increasing darkness and the rostrum protrudes anterior to the mouth [23]. During this period, caudal denticles develop from posterior to anterior in dorsal and ventral rows, on either side of the tail tip. At early Stage 27, no placodes can be detected (*a2*–*a2*). Epithelial thickenings then form from posterior to anterior (*b2*–*b3*, *c2*–*c4*). *c4* shows a magnified view of *c3*, highlighting an individual placode (marked with an *arrowhead*). These placodes then accumulate their first layers of mineralised tissue during morphogenesis (*d2*–*d4*). *d4* shows a magnified view of *d3*, highlighting a mineralising placode (marked with an *arrowhead*). Mineralisation of denticles also occurs sequentially from posterior to anterior (*e2*–*e3*, *f2*–*f3*, *g2*–*g3*, *h2*–*h3* and *i2*–*i3*) and can be highlighted with alizarin red staining for calcium-rich tissue (*e4*–*e5*, *f4*–*f5*, *g4*–*g5*, *h4*–*h5* and *i4*–*i5*). For the axis, *D* dorsal, *V* ventral, *P* posterior and *A* anterior. *Scale bars* are 1000 µm for *a1*, *b1*, *c1*, *d1*, *e1*, *f1*, *g1*, *h1* and *i1* and 200 µm for all other images

**Fig. 3:**
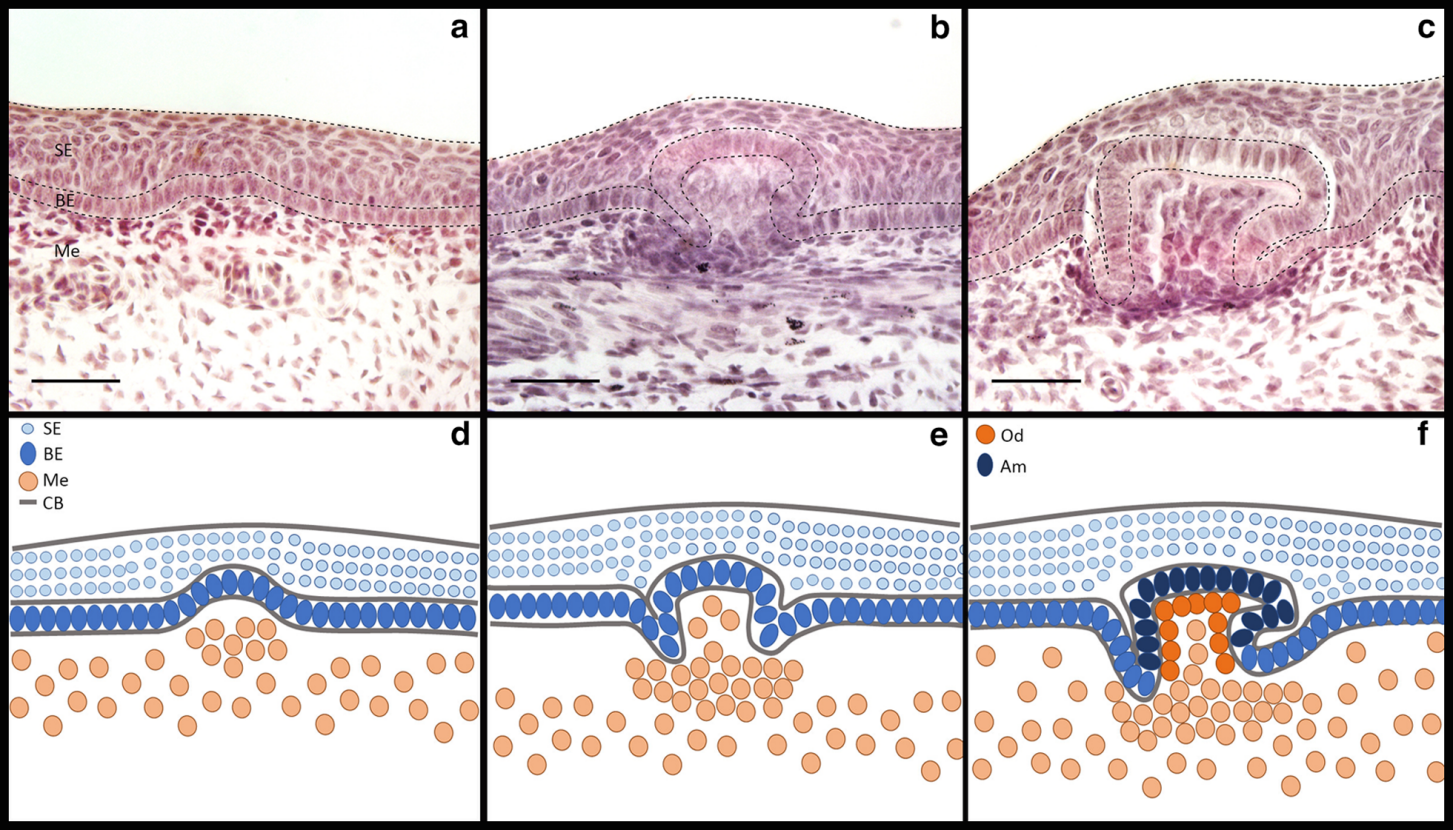
Morphogenesis of a caudal denticle. Caudal denticle placodes consist of a squamous epithelium (SE) overlying columnar cells of the basal epithelium (BE), which overlies the mesenchyme (Me) (*a*, *d*). During placode morphogenesis, condensing mesenchymal cells aggregate below columnar cells of the basal epithelium epithelial. The basal epithelium undergoes growth and folding (*b*, *e*) to form the posterior facing cusp (*c*, *f*). CB is cell layer boundary. Ameloblasts (Am) in the basal epithelial cusp (*c*, *f*) and odontodes (Od) in the papilla underlying the basal epithelium produce enameloid and dentine, respectively, to mineralise the unit [40]. *Scale bars* are 50 µm

To determine the earliest time of caudal denticle morphogenesis in the shark (*S. canicula*), we charted the sequential development of these units. It has been documented that caudal denticles in *S. canicula* develop from a posterior to anterior direction in dorsal and ventral rows, on both sides of the caudal-most tip of the tail (Fig. 2) [23, 27]. Previous reports suggest their emergence occurs at 52–60 dpf (Stage 30) [23] (Fig. 2f, g). However, our observations suggest placode development begins earlier, between 42 and 46 dpf (Stage 27) [23] (Fig. 2b, c), although some variation in timing of denticle initiation was noted. One explanation for such variation in development is temperature of the surrounding environment [23]. Caudal denticles arise from distinct placodes (Figs. 2c4, 3a), which form from a thickened condensation of epithelial cells with an underlying mesenchymal condensate (Fig. 3). The first denticle placodes to form (most posterior) are also the first in the sequence to mineralise. This progresses in a posterior–anterior fashion (Fig. 2d4) and can be visualised using alizarin red staining (Fig. 2e4–i5).

The sequential development of this morphogenetic placode unit bears remarkable similarity to feather bud development in chicks [4, 36, 47, 48] (Fig. 2). We wished to test whether members of the amniote epithelial appendage placode GRN are conserved in chondrichthyans [4, 8]. Therefore, a selection of well-known GRN components assembled from the literature regarding feather, hair and tooth development were chosen [36, 49, 50], and their expression during early placode morphogenesis of caudal denticles in *S. canicula* was examined.

### Gene expression from integumentary appendage development is conserved in sharks

**Fig. 4:**
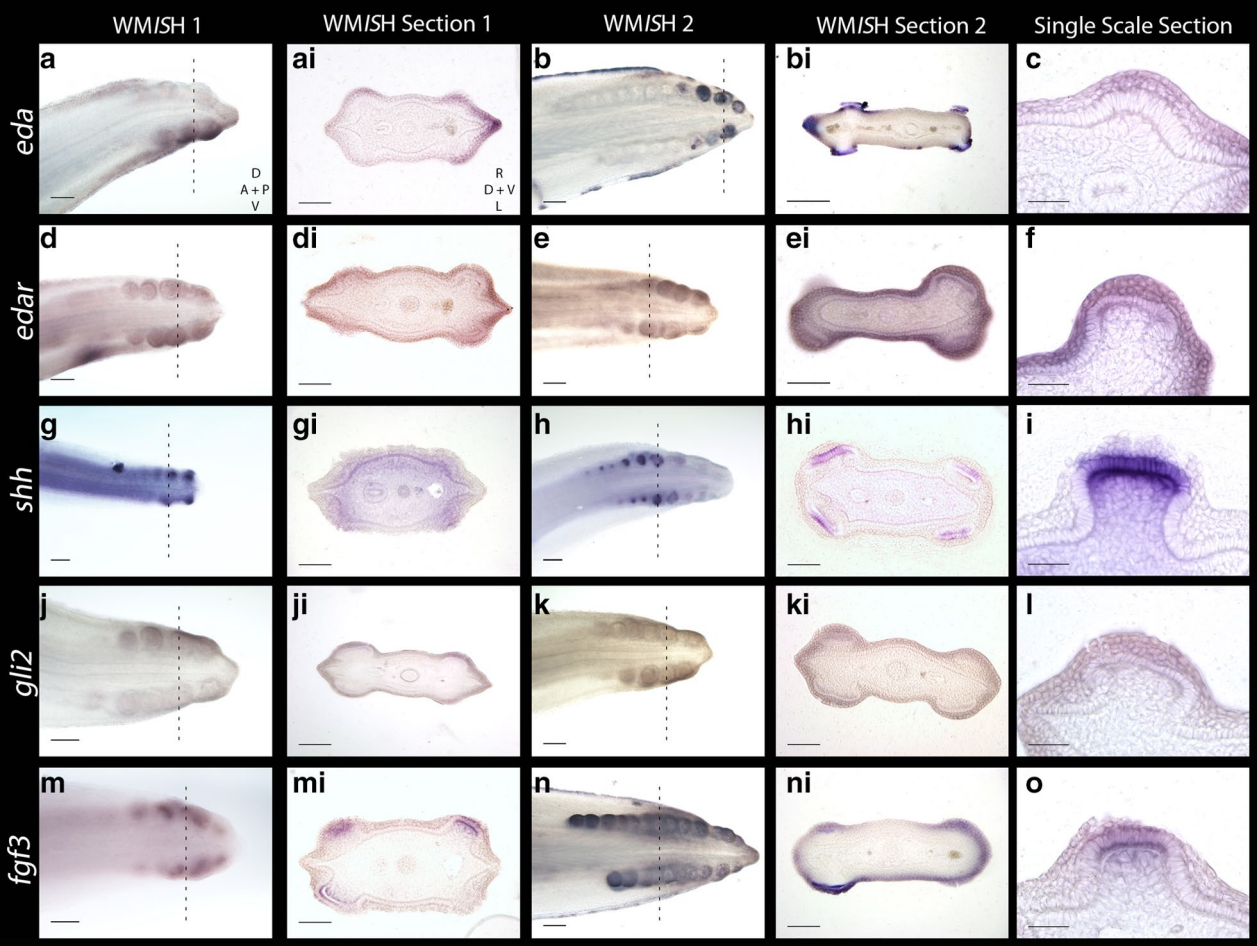
Gene expression analyses of early morphogenesis of caudal denticles. Expression of *eda* and its receptor *edar* are observed in the epithelium during early placode morphogenesis (*a*–*f*). *eda* can also be seen in tissue undergoing mineralisation later in morphogenesis (*b*–*bi*). *shh* is first observed in the epithelium during early morphogenesis, before becoming restricted to the basal epithelium later in morphogenesis (*g*–*i*). *gli2* is also seen in the epithelium early during placode formation (*j*–*l*). Expression of *fgf3* is first seen in the epithelium, before moving to both the epithelium and mesenchyme later in placode morphogenesis (*m*–*o*). The *dashed lines* show where in the WM*IS*H the section was taken. WM*IS*H Section 1 represents a younger stage specimen than WM*IS*H Section 2. For the WM*IS*H, *D* dorsal, *V* ventral, *A* anterior and *P* posterior. For WM*IS*H sections, *R* right, *L* left, *D* dorsal and *V* ventral. For *scale bars*, *a*, *b*, *d*, *e*, *g*, *h*, *j*, *k*, *m*, *n* = 200 µm, *ai*, *bi*, *di*, *ei*, *gi*, *hi*, *ji*, *ki*, *mi*, *ni* = 100 µm, and *c*, *f*, *i*, *l*, *o* = 50 µm

Recent research has revealed ectodysplasin signalling is conserved throughout development of amniote epithelial appendages [4]. Ectodysplasin-A (*Eda*) and its receptor (*Edar*) comprise some of the earliest markers of placode morphogenesis in vertebrates (including zebrafish, chick and mouse) [49, 51–54]. During early morphogenesis of shark caudal denticles, *eda* and *edar* expression is detected in the localised epithelial thickening (Fig. 4a–f). *eda* is also expressed during later denticle morphogenesis in epithelial cells in the signalling centre of the putative enameloid knot (EK) (Fig. 4b, bi). This shares similarity to mammalian tooth development, during which interactions between Eda/Edar and other signalling molecules (e.g. Shh, Fgf4 and Bmp4) regulate morphogenesis of the enamel knot [55]. During hair morphogenesis in mammals, Eda and Edar signalling induces expression of other signalling molecules, such as Shh [56, 57].

Shh is a ligand of the Hedgehog (Hh) signalling pathway that marks early stages of epithelial morphogenesis in a diverse range of integumentary organs [50, 58, 59], including shark teeth and chick feathers [14, 40, 59, 60]. *shh* is expressed throughout morphogenesis of shark caudal denticles (Fig. 4g–i). During early morphogenesis, *shh* is first expressed in the superficial squamous epithelium (Fig. 6m), before subsequently becoming restricted to the basal epithelium (Figs. 6n, o, 7). Previous research has shown that *Gli2* is expressed both downstream and upstream of Shh signalling [61–63] and is essential in hair follicle development as a promoter of cell proliferation [64]. Here, we found that *gli2* is also expressed in the epithelial cells of developing placodes (Fig. 4j–l).

**Fig. 5:**
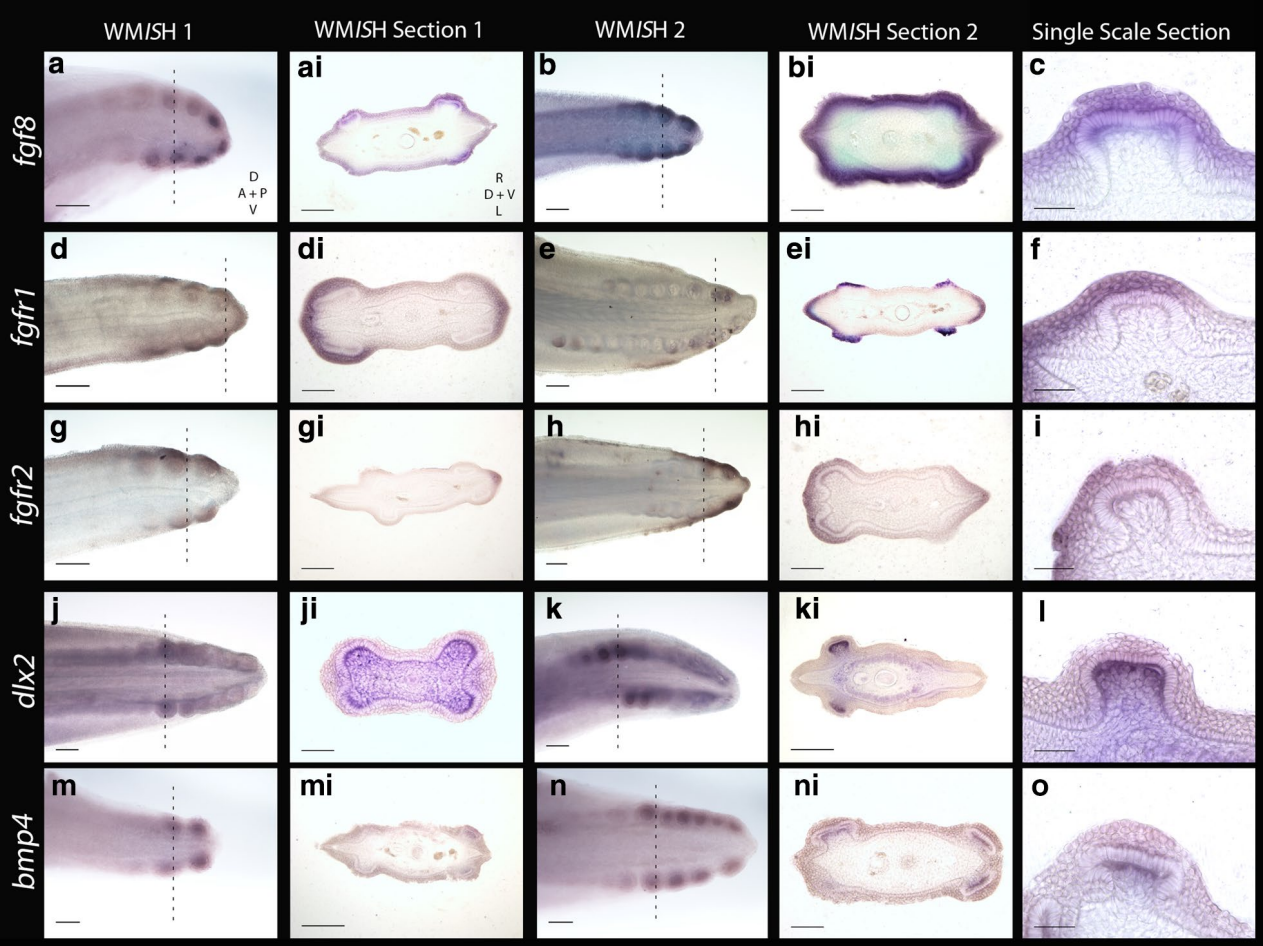
*fgf8* signalling is largely retained in the epithelium throughout (*a*–*c*). *fgfr1* and *fgfr2* are both seen in the epithelium during early denticle morphogenesis (*d*–*i*). Expression of *dlx2* is restricted to the mesenchyme throughout early placode morphogenesis (*j*–*l*). Similarly, *bmp4* is observed in the mesenchyme during early placode morphogenesis (*m*–*o*). The *dashed lines* show where in the WM*IS*H the section was taken. WM*IS*H Section 1 represents a younger stage specimen than WM*IS*H Section 2. For the WM*IS*H, *D* dorsal, *V* ventral, *A* anterior and *P* posterior. For WM*IS*H sections, *R* right, *L* left, *D* dorsal and *V* ventral. For *scale bars*, *a*, *b*, *d*, *e*, *g*, *h*, *j*, *k*, *m*, *n* = 200 µm, *ai*, *bi*, *di*, *ei*, *gi*, *hi*, *ji*, *ki*, *mi*, *ni* = 100 µm, and *c*, *f*, *i*, *l* and *o* = 50 µm

**Fig. 6:**
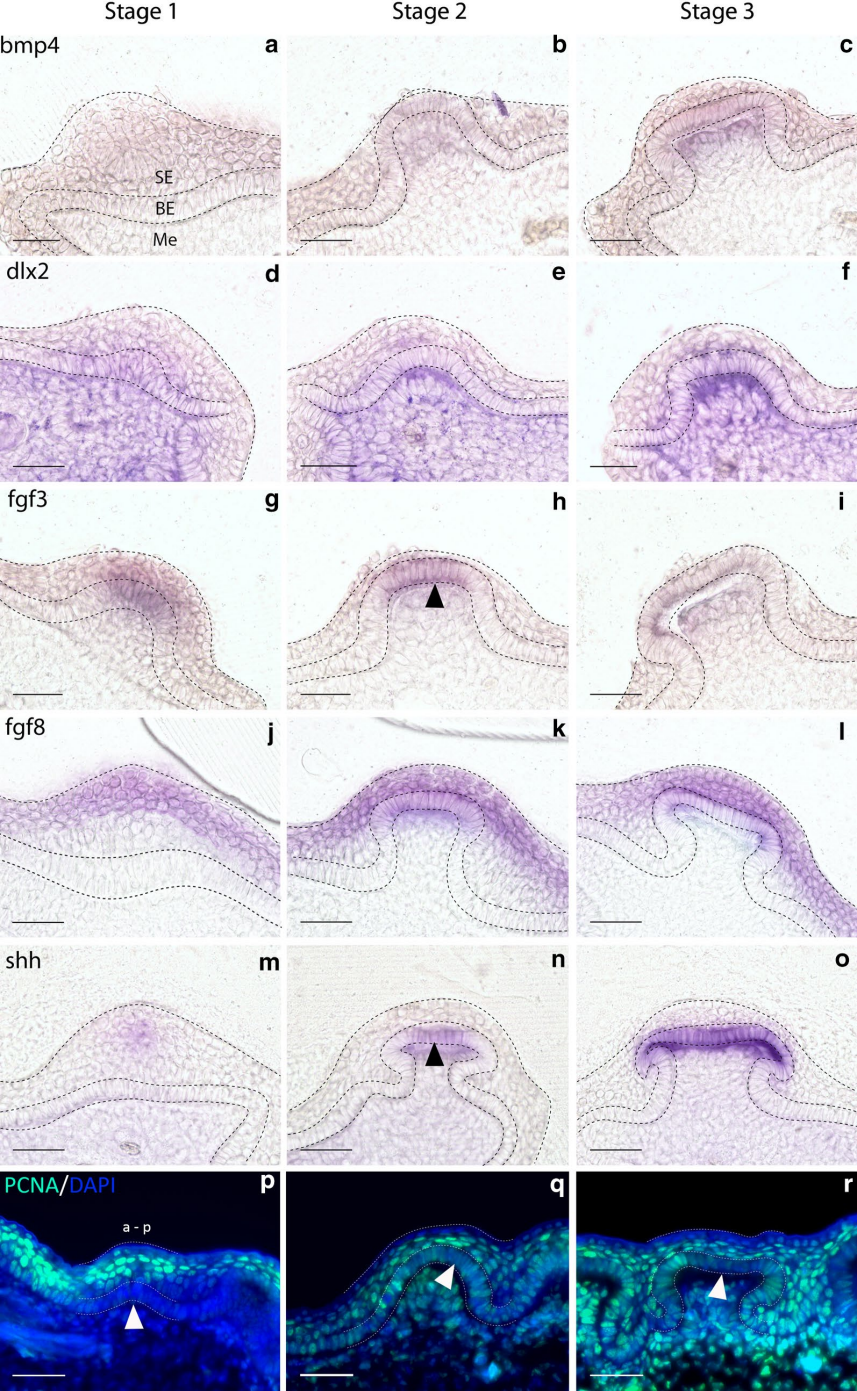
Gene expression/PCNA analysis of early caudal denticle morphogenesis. Gene expression is shown in 30-µm transverse sections of wild-type *S. canicula* embryo tails post-WM*IS*H, to highlight progressive stages of caudal denticle morphogenesis from the initial epithelial thickening. *bmp4* and *dlx2* expression is restricted to the mesenchyme throughout morphogenesis (Me) (*a*–*f*). *shh* and *fgf8* are first observed in the squamous epithelium (SE) before becoming restricted to the basal epithelium (BE) (*m*–*o*, *j*–*l*). Expression of *fgf3* begins in the squamous and basal epithelium and is subsequently observed throughout the epithelium and mesenchyme (*g*–*i*). PCNA immunofluorescence is observed in the epithelium and mesenchyme throughout morphogenesis (*p*–*r*). Reduced activity (marked with an *arrowhead*) was noted in columnar cells of the epithelium during early morphogenesis (*p*) and in a central region of columnar cells of the basal epithelium during later morphogenesis (*q*–*r*). This region (*q*) overlaps with *fgf3* and *shh* expression in the basal epithelium (*k*, *n*) (marked with an *arrowhead*) and may be indicative of a basic primary enameloid knot. a is anterior, and p is posterior. *Dashed lines* separate the squamous epithelium (SE), basal epithelium (BE) and mesenchyme (Me). All *scale bars* are 50 µm in length

**Fig. 7:**
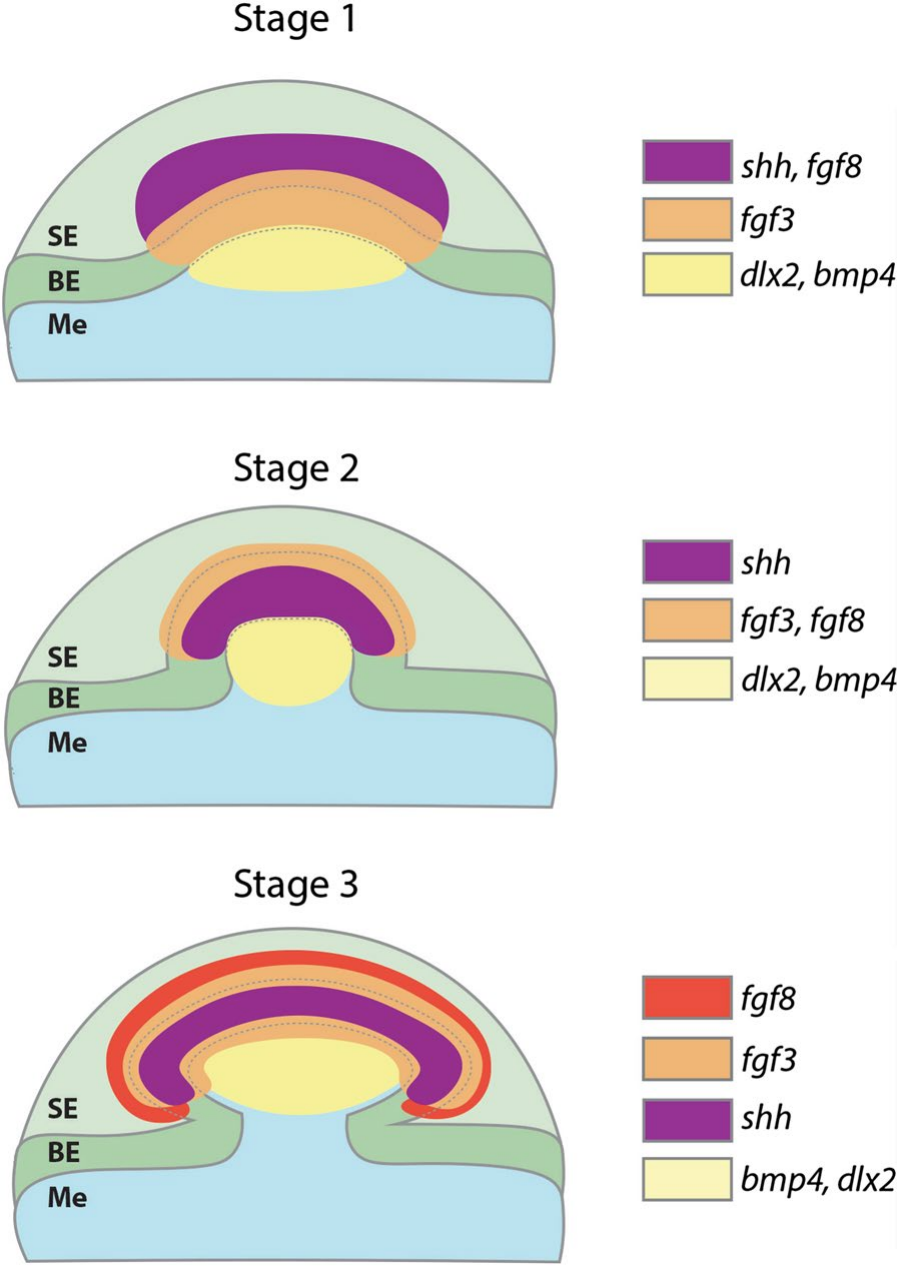
Schematic diagram representing gene expression during early morphogenesis of caudal denticles. This diagram summarises the results from Figs. 4 and 5, representing expression of *fgf3*, *fgf8*, *shh*, *bmp4* and *dlx2* throughout progressive stages of early morphogenesis. SE is the squamous epithelium, BE is the basal epithelium and Me is the mesenchyme

In various aspects of vertebrate appendage development, Shh and FGFs (Fgf4, Fgf8) exhibit interdependent positive feedback loops that promote the expression of either molecule [65, 66]. *Fgf3* expression is mesenchymal during early morphogenesis of both feathers [67] and teeth, although in later tooth morphogenesis it is present in the epithelium of the primary enamel knot [68]. In shark caudal denticles, *fgf3* expression is initially epithelial, although it is later seen in both the epithelium and mesenchyme, in a pattern similar to shark tooth and body denticle development [14, 40] (Figs. 4m–o, 6g–i, 7). Fgf8 is an epithelial initiatory signal of mammalian tooth morphogenesis [69]. In the shark, *fgf8* expression is observed in the epithelium during early caudal denticle morphogenesis, at a similar stage to *shh* (Figs. 5a–c, 6j–l, 7), and remains in the epithelium during later morphogenesis of the denticle cusps (Fig. 5l).

Studies from teleosts (medaka) and mammals (mouse) have indicated that Fgf8 is a ligand of Fgfr1 [70–72] that regulates enamel formation during mammalian tooth morphogenesis [73]. During early caudal denticle morphogenesis, we observed expression of *fgfr1* in the squamous epithelium of placodes (Fig. 5d–f). *fgfr1* is also expressed throughout the epithelium later in morphogenesis during caudal denticle mineralisation (Fig. 5e–ei), which may be indicative of a conserved role regulating enameloid formation. Fgfr2 can transduce Fgf3 during mammalian development [74, 75]. During early morphogenesis of caudal denticles, *fgfr2* is expressed in the squamous epithelium of the early developing placodes in *S. canicula* (Fig. 5g–i). This pattern is similar to epithelial expression of *fgf3*, suggesting the role of fgfr2 as a fgf3 signal transducer could be conserved.

*Dlx2* is a member of the Dlx homeodomain transcription factor family, which is widely important throughout various aspects of vertebrate development, including epithelial appendage formation [76, 77]. Fgf8 regulates *Dlx2* expression in the underlying mesenchyme during both mouse tooth and branchial arch development [78, 79]. Previously, *dlx* gene expression has been documented during caudal denticle morphogenesis in *S. canicula* [41]. Our results confirm *dlx2* is expressed in caudal denticles, and additionally we show that expression is restricted to the mesenchyme throughout early morphogenesis (Figs. 5j–l, 6d–f, 7), as observed during mouse tooth development [78].

Mesenchymal Bmp4 has an inhibitory role during amniote epithelial appendage development [1, 36, 46]. Consistent with expression observed during feather, shark tooth and body denticle development [14, 36, 46], *bmp4* is expressed in the mesenchyme during early morphogenesis of caudal denticle placodes (Figs. 5m–o, 6a–c, 7). bmp4 may also be acting as an internal inhibitor here [1, 36], helping to define the size of the placode and therefore the adult caudal denticle.

**Fig. 8:**
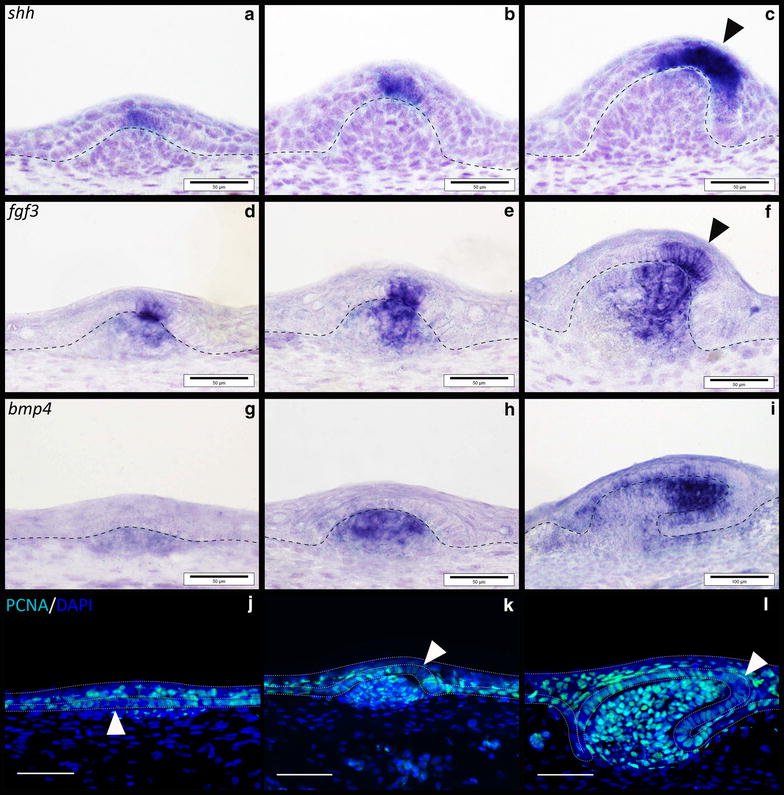
Gene expression analysis of putative placode GRN members, during general body denticle development. Section in situ hybridisation (S*IS*H) was undertaken during early development of body denticles. Expression of *shh* was epithelial throughout development (*a*–*c*), whereas *fgf3* was observed in both the epithelium and mesenchyme (*d*–*f*). *bmp4* was mesenchymal throughout early morphogenesis (*g*–*i*). PCNA immunoreactivity was observed in the epithelial cells and condensing mesenchyme of emerging denticles (*j*–*l*). Reduced immunoreactivity was noted in columnar cells of the basal epithelium during placode formation (*j*) (*white arrowed*). *fgf3* and *shh* expression marks enameloid knot-like cells of the epithelium associated with denticle morphogenesis (*c*, *f*), which also show reduced PCNA immunoreactivity (*l*), characteristic of this signalling centre (*black arrowheads*). The *dashed line* separates the epithelium from the underlying mesenchyme (*a*–*i*), as well as the basal epithelium and squamous epithelium (*j*–*l*). All *scale bars* are 50 µm in length except for image *i* for which the *scale bar* is 100 µm

In addition to examining caudal denticle development, gene expression of these putative core GRN members was also examined in general body denticles to compare signalling between different odontode types (Figs. 1, 8). Expression of *shh* is restricted to the epithelium throughout early morphogenesis of general body denticles (Fig. 8a–c), whereas *fgf3* is first observed most strongly in the epithelium (Fig. 8d) before being expressed in both the epithelium and underlying mesenchyme (Fig. 8e, f). Epithelial *fgf3* overlaps with *shh* expression in the putative enameloid knot, whereas *bmp4* is restricted to the mesenchyme throughout morphogenesis (Fig. 8g–i). These results show conservation of gene expression patterns between caudal and general body denticles.

Proliferating cell nuclear antigen (PCNA) immunoreactivity marks several phases of cell division from late G1 to mitosis [80]. Developing body denticles are highly proliferative units similar to teeth and dental lamina [40, 42] (Fig. 8j–l). During early morphogenesis, the columnar cells of these placodes are characterised by reduced proliferation (Fig. 8j), as also observed throughout amniote skin appendage development [4]. Interestingly, a region of the apical denticle cusp also shows marked reduction in PCNA immunoreactivity (Fig. 8k, l) that corresponds to a putative signalling centre comparable to the enameloid knot in shark teeth [40]. This set of cells appears to overlap with the region of *shh* and *fgf3* expression in the polarised cells that will become the apical cusp (Fig. 8a–f). Caudal denticles display comparable PCNA immunoreactivity during morphogenesis (Fig. 6p–r), including reduced proliferation of columnar cells during early morphogenesis compared to younger anterior epithelial tissue in which placode formation has not begun (Fig. 6p). However, the region of reduced proliferation that occurs in columnar epithelial cells later during morphogenesis appears to be positioned more centrally than observed in body denticles, which have a distinct polarity (Fig. 6q, r). This region overlaps with expression of *fgf3* and *shh* (Fig. 6k–n), and could also be indicative of a putative primary enameloid knot, as observed in general body denticles (Fig. 8) [40]. The positional variation of this enameloid knot could reflect a shift in the morphology of these units, as caudal denticles display a less definitively polarised cusp than general body denticles (Fig. 1).

In the absence of functional data, it is not possible to test for the conserved action of GRN members, which could yield important clues regarding the putative homology of denticles and amniote epithelial appendages. We have therefore initiated a small-molecule-based targeted signalling pathway-knockdown screening assay in *S. canicula* to test the function of putative epithelial appendage GRN members, based on published results from other vertebrates.

### Small molecule inhibition reveals dependency of caudal denticle development on FGF signalling

**Fig. 9:**
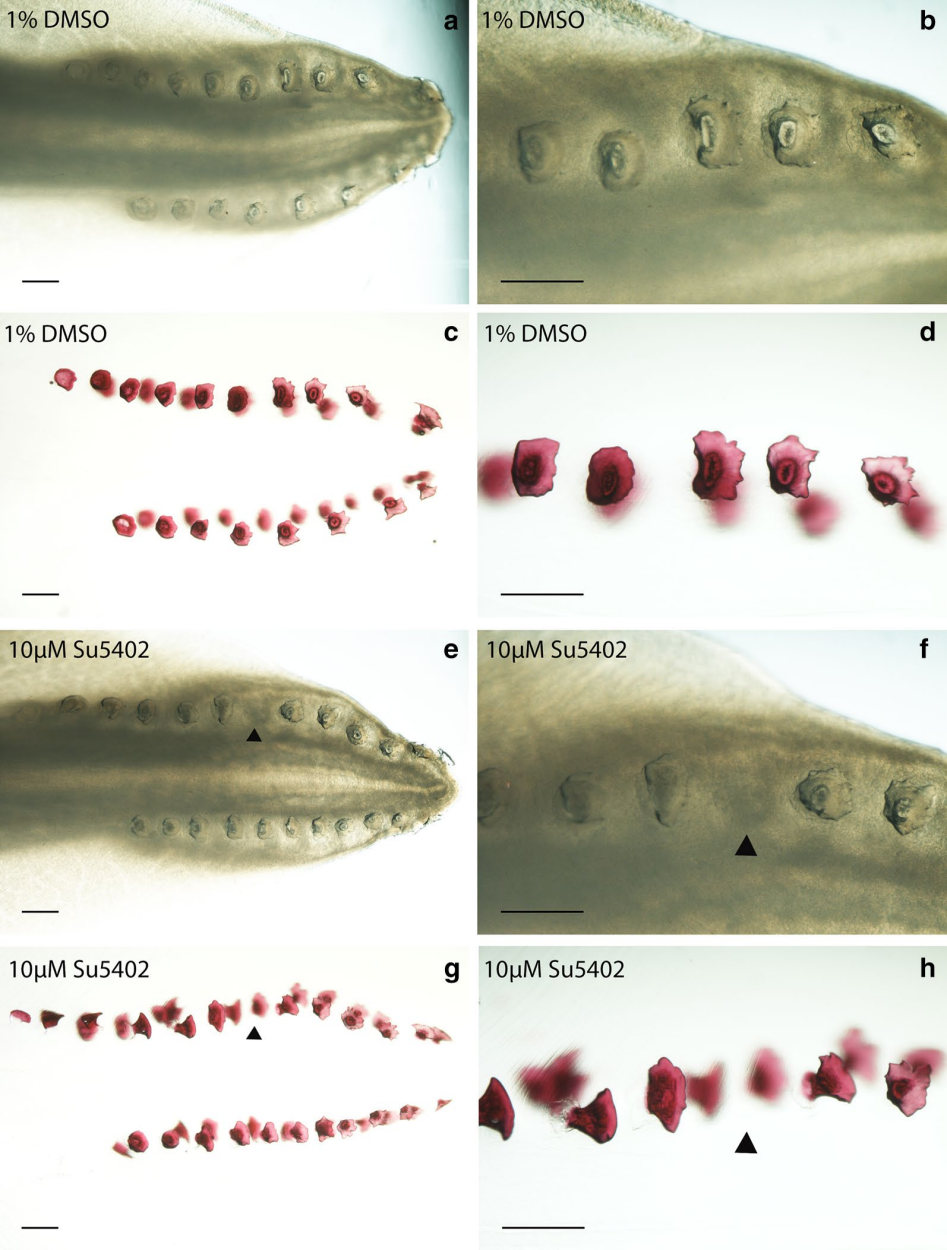
Phenotypic effect of FGF inhibition via SU5402 treatment (10 µM) on caudal denticle development. The DMSO control specimen shown after fixation (*a*, *b*) and cleared and stained for calcium-rich tissue using alizarin red (*c*, *d*) possesses a full sequence of caudal denticles. However, the specimen treated with the FGF antagonist SU5402 has the 6th denticle missing from the sequence, shown after fixation (*e*, *f*) and cleared and stained (*g*, *h*). This is marked with a *black arrowhead*. This denticle knockout corresponds to the stage at which treatment occurred, and was observed in 40% of Su5402-treated specimens (*n* = 5). *Scale bars* are 200 µm in length

To elucidate the specific roles of FGF signalling during early caudal denticle placode morphogenesis, in vivo pathway perturbation assays were undertaken using SU5402. This chemical inhibits FGF signalling by blocking FGFR activity [81–83]. Stage 28 *S. canicula* embryos were treated in their sealed egg cases by injection with SU5402 to a final concentration of ~10 µM for 25 days and then allowed to develop for a further 35 days following the opening of their egg cases and washing with fresh artificial seawater. Treatment with SU5402 resulted in a single denticle knockout in 40% of the treated samples (*n* = 5) and none of the DMSO-treated control samples (*n* = 5) (Fig. 9). These units normally form equidistant from each other [23, 28, 29]; however, in the drug treated specimen shown (Fig. 9e–h) the 6th denticle was missing in the left-side dorsal row. This corresponds to the time at which treatment took place, when approximately 5 caudal denticle placodes had developed in sequence on each row (Fig. 2c). Therefore, ~10 µM SU5402 appears to prevent placode formation and subsequent morphogenesis, indicating this process is dependent upon FGF signalling. As only a single denticle was lost from the sequence, it is likely the chemical either diffused out of the egg case or decomposed within it after its initial inhibitory action. We observed a similar result in our preliminary SU5402 treatment trial (see Additional file 1), which revealed a vestige when stained with alcian blue, indicative of denticle abortion. The relatively short window of sensitivity to FGF inhibition by SU5402 treatment coupled with the offset in developmental timing of individual caudal denticle rows is likely to provide an explanation for the unilaterality of this denticle knockout. As subsequent placodes developed, the field of initiatory competence is likely to have already been in place, enabling the sequential, iterative patterning to proceed beyond the disturbance once the effect of SU5402 had subsided.

We sought to confirm a specific effect of SU5402 upon the FGF signalling pathway and the placode-forming GRN by examining expression of participating network members. The prior assay required longer-term development of embryos to observe morphological effects (60 days) and subsequently used a lower drug concentration to avoid mortality, as this was an issue in preliminary trials. Experimental perturbation of FGF signalling was therefore repeated using a higher concentration of SU5402 (1 × 50 µM injection every 24 h, over a 96-h period), in line with previously published assays [37, 84, 85], and embryos were fixed immediately after the 96-h period. Specimens were then processed for in situ hybridisation for a selection of the same putative GRN members examined previously in wild-type embryos (Figs. 4, 5, 6). This allowed us to test whether perturbation of FGF signalling in shark denticles disrupted other members of the placode GRN in a manner consistent with a conserved relationship between network members.

**Fig. 10:**
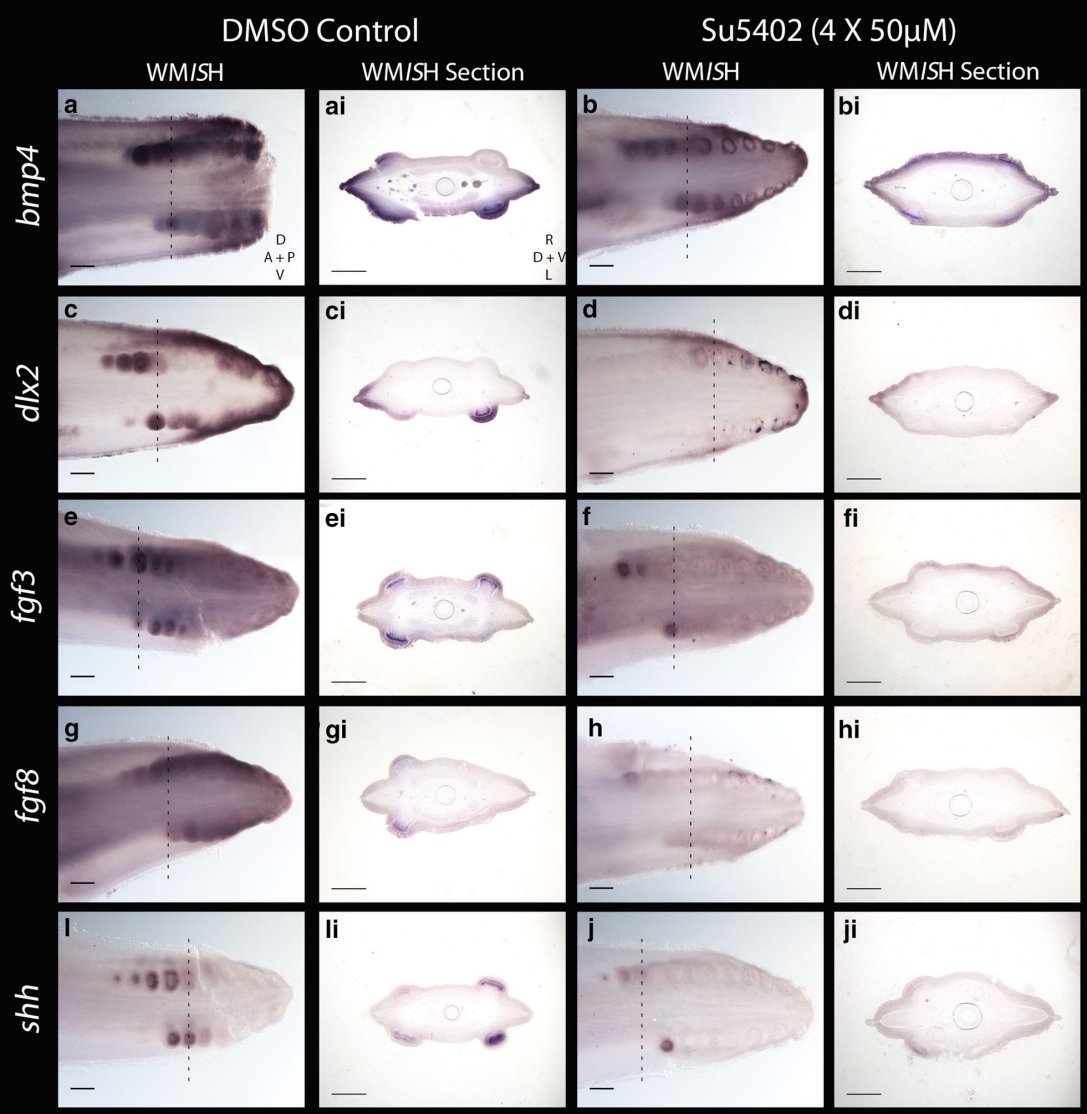
Genetic effect of FGF inhibition via SU5402 treatment (4 × 50 µM) on caudal denticle development. There was a reduction in staining intensity of *bmp4* (*a*–*bi*), *dlx2* (*c*–*di*), *fgf3* (*e*–*fi*), *fgf8* (*g*–*hi*) and *shh* (*i*–*ji*) in SU5402-treated specimens compared to DMSO-treated controls. We propose this resulted from the interruptions to the following GRN interactions. SU5402 inhibits FGF activity by blocking FGFR activity, thereby reducing expression of *fgf3* and *fgf8* (*e*–*hi*). This reduced *shh* and *dlx2* expression as a FGF—*shh* positive feedback loops that would normally promote *shh* and *dlx2* expression (as observed during feather development) were interrupted (*i*–*ji*, *c*–*di*) [78]. The *fgf4*–*shh* positive feedback loop that promotes *bmp4* was also interrupted by the SU5402 treatment, reducing *shh* and *bmp4* expression (*i*–*ji*, *a*–*bi*) [36]. SU5402-treated and DMSO control specimens both underwent the colour reaction of the WM*IS*H protocol for the same length of time. The *dashed lines* show where the section was taken from. *Scale bars* for WM*IS*H are 200 µm in length, and for the WM*IS*H sections they are 100 µm in length

FGF ligands exhibit positive feedback loops with Shh during many aspects of vertebrate embryogenesis, including epithelial appendage, limb bud and gill arch development [36, 65, 66, 86, 87]. We observed a dramatic downregulation of *fgf3*, *fgf8*, *shh* and *dlx2* expression in the SU5402-treated individuals compared the DMSO-treated controls, in all but the youngest (most anterior) denticles (Fig. 10c–ji). Expression intensity of *bmp4* was also notably reduced compared to the control (Fig. 10a–bi). Two SU5402-treated specimens were used for WM*IS*H for each marker, along with one DMSO control specimen. These results suggest that SU5402 blocked FGF/FGFR signalling [81–83], thereby reducing expression of *fgf3* and *fgf8* (Fig. 10e–hi). This is likely due to SU5402 blocking earlier FGF signalling required for expression of these ligands [37], and interrupting the FGF–Shh positive feedback loop, which consequently limited expression of *shh*, *bmp4* and *dlx2* (Fig. 10a–di, i–ji) [36, 66, 78, 85]. Dlx family members have a role downstream of FGFs during feather bud development [77], indicating that this downregulation of *dlx2* is likely a result of FGF inhibition. These results suggest that during caudal denticle formation, the function of FGF signalling in the GRN which guides epithelial appendage morphogenesis is conserved between sharks and other vertebrates.

## Discussion

### An FGF-dependent GRN constructs the placodes of epithelial appendages throughout jawed vertebrates

Our results suggest that a conserved core GRN, which includes *eda*/*edar*, *shh*, *gli2*, *fgf3*, *fgf8*, *bmp4* and *dlx2*, underlies the development of epithelial integumentary appendage placodes across jawed vertebrates (Figs. 4, 5, 6). These placodes possess columnar epithelial cells with a reduced rate of proliferation (Fig. 6p), which is considered a structural characteristic of amniote skin appendage development [4]. Functional experiments revealed that expression of these GRN members is influenced by the FGF signalling pathway and that normal denticle development is perturbed upon inhibition with SU5402 (Figs. 9, 10). Caudal denticles are considered an ancient epithelial appendage that may have originated in early vertebrates over 450 million years ago and have been retained in some extant chondrichthyans [10, 11, 27]. The historical continuity of the anatomical placode and underlying GRN in both amniotes [4] and chondrichthyans provides evidence for the historical homology of all vertebrate epithelial appendages [88].

Previously, researchers have speculated that epithelial appendages have evolved independently in mammals, reptiles and birds and that therefore molecular similarity of GRNs could be a result of independent genetic co-option or deep homology [9, 89–92]. However, recent evidence has suggested that integumentary epithelial appendages are historically homologous, at least throughout all amniotes on the basis of the anatomical placode with conserved expression and function of GRN members [4]. Our results suggest this historical homology extends even further into vertebrate phylogeny and may encompass the integumentary epithelial appendages of all extant jawed vertebrates.

**Fig. 11:**
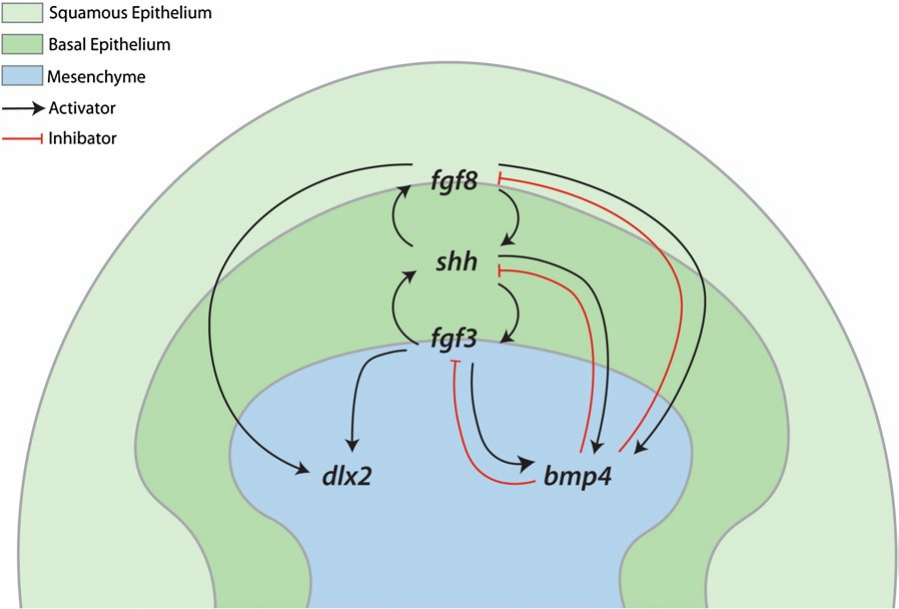
Putative relationship between FGF and associated GRN components during caudal denticle morphogenesis. As observed widely throughout epithelial appendage development, for example during feather placode development, FGF—*shh* positive feedback loops which promote mesenchymal *bmp4* are likely to promote early caudal denticle placode morphogenesis. *bmp4* may then act as an internal inhibitor, limiting the size of the final unit. FGF signalling can also promote mesenchymal expression of *dlx2*. This is a hypothetical GRN based on findings from previous research, gene expression data (Figs. 4, 5, 6, 8) and small molecule inhibition of FGF signalling during early caudal denticle morphogenesis, using SU5402 (Figs. 9, 10)

During both mouse and zebrafish tooth morphogenesis, Fgf8 signalling can promote *Dlx2* expression [37, 78, 79]. Inactivation of *Fgf8* can result in both misregulation of *Fgf4* and *Shh* [87], which are also known to work together in autocatalytic positive feedback loops during vertebrate development, for example in limb and feather patterning [36, 65]. During early feather placode morphogenesis, this Shh–Fgf4 feedback loop promotes *Bmp4* expression, which subsequently acts as an inhibitor to limit their expression in a negative feedback loop [36]. Our results regarding gene expression of FGF-perturbed shark embryos reveal this functional conservation likely extends to the denticles of sharks (Figs. 10, 11).

Despite the broad conservation of this GRN, our observations of gene expression patterns did highlight some taxonomic disparity. We showed that *fgf3* is expressed in both the epithelium and later the mesenchyme of denticle placodes, as observed during mouse and shark tooth development [40, 68]. This contrasts with known expression patterns observed during zebrafish pharyngeal tooth and chick feather development [37, 67]. Similarly, *Fgf8* is an important inductive signal during mammalian tooth development [69] and is present during caudal denticle development, but is absent from feather or zebrafish tooth morphogenesis [33, 37]. There is potential for evolutionary alterations to gene expression and functionality throughout the FGF signalling family, and paralogs may perform the same developmental role in different taxa in a process known as function shuffling [93], for example mammalian *Fgf8* may have a zebrafish specific paralog [37].

Recent research has revealed chondrichthyan general body denticles and teeth are deeply homologous developmental units, despite differences in their regenerative capacities [14, 40]. Our findings suggest this odontode GRN additionally encompasses caudal denticles, as conserved expression patterns were observed throughout early placode morphogenesis between caudal denticles and general body denticles. Caudal denticles are morphologically disparate from general body denticles, dorsal denticles and teeth (Fig. 1), and positional alterations to the putative enameloid knot may contribute to this variation (Figs. 6, 8). As predicted by an hourglass model of development [94], divergence in the GRN later in morphogenesis is also likely to result in alterations to the adult form, constructing different structures upon a homologous foundation: the anatomical placode. Such divergence of networks is known to relate to variation in the adult structure of feathers and teeth [95, 96].

## Conclusion

The early morphogenesis of vertebrate epithelial appendages is likely to be a universal and highly conserved process retained over evolutionary time and modified to form the plethora of diverse skin appendages observed throughout all vertebrates, from sharks to mammals. The placodes of vertebrate epithelial appendages constitute the conserved foundations upon which integumentary structures have evolved, via alterations to an otherwise conserved GRN that take effect during later morphogenesis. The shark caudal denticle system provides an ideal set of sequentially developing integumentary epithelial appendages that can be studied further to decipher both complex functional GRNs and patterning mechanisms.

Combining techniques such as small molecule signalling pathway perturbation with gene expression analyses can help us begin to interpret the roles of putative GRN members. The set of genes investigated here were chosen due to their importance in the development of other epithelial appendages; however, there are many important molecules and interactions to further investigate, for example those associated with the Wnt/β-catenin and Notch pathways [97, 98]. A focus on investigating downstream GRN components responsible for later morphogenesis will enable us to elucidate how historically homologous placodes develop into the diverse range of epithelial appendages observed throughout vertebrates.

## Methods

### Shark husbandry and fixation

The University of Sheffield is a licensed establishment under the Animals (Scientific Procedures) Act 1986. All animals were culled by approved methods cited under Schedule 1 to the Act. Embryos were imported from ‘Station Biologique’ in Roscoff, France, and housed in tanks at The University of Sheffield, Animal and Plant Sciences, at 16 °C. Salinity was adjusted to replicate sea water using ‘Instant Ocean’ salt dissolved in dechlorinated water. Water was oxygenated with a submerged airflow. 50% water changes were undertaken on a weekly basis. Embryos were removed from their egg cases, anesthetised using MS-222 (Tricaine) and fixed overnight at 4 °C in 4% paraformaldehyde (PFA). Samples were dehydrated through a graded series of PBS and MeOH and stored at −20 °C in MeOH.

### Alizarin red clear and staining

Fixed specimens were rehydrated through a graded series of MeOH and PBS. Staining took place in darkness overnight in 0.01% alizarin red dissolved in 0.5% KOH. Specimens were treated with trypsin in saturated sodium borate and distilled water. For Additional file 1, the sample was stained with 0.1% alcian blue in EtOH and acetic acid before the alizarin red stain was applied. Samples were then run through a graded series of KOH and glycerol solutions, before imaging took place in glycerol, using a Nikon SMZ1500 stereomicroscope.

### Haematoxylin and eosin (H&E) staining

Paraffin-embedded sections were deparaffinised in xylene and rehydrated through a graded series of MeOH and PBS, before staining with haematoxylin. Sections were then rinsed in ddH_2_0, washed with HCl in EtOH and washed with 0.001 M Tris–HCL. Finally, sections were stained with eosin, dehydrated to MeOH and mounted used DePeX mounding medium (VWR). Samples were imaged using an Olympus BX51 microscope and Olympus DP71 Universal digital camera attachment.

### Micro-computerised tomography (MicroCT) and light sheet fluorescence microscopy (LSFM)

High-resolution MicroCT scanning was carried out upon a Stage 32 embryo stained with 0.1% PTA (phosphotungstic acid) in 70% EtOH for 3 days, using an Xradia MicroXCT scanner at the Imaging and Analysis Centre of the Natural History Museum (London). Scans were rendered using the 3D volume exploration tool Drishti (www.github.com/nci/drishti) (Fig. 1j, k). LSFM was carried out upon alizarin-stained samples. A Zeiss Z1 light sheet microscope with two sCMOS cameras and an acquisition PC running Zen Black 2014 software was used to scan the tail of a Stage 31 embryo. Rendering was undertaken using the image analysis software Imaris (www.bitplane.com/imaris/imaris) by creating a signal intensity-based isosurface (Fig. 1l, m).

### Small molecule gene perturbation experiments

For the first SU5402 treatment trial, Stage 28 *S. canicula* embryos [23] were treated with the FGF-receptor inhibitor SU5402 (Sigma). At this stage, the egg case is sealed from the external environment, allowing administration of drugs via injection into the vitelline fluid. The egg case acts as a natural treatment chamber. 100 µl of a 500 µM stock solution of SU5402 in 1% DMSO in PBS was injected into 5 egg cases, to achieve a ~10 µM concentration of SU5402 assuming an approximate egg case size of 5 ml. 5 control samples were treated with 100 µl of 1% DMSO in PBS. At Stage 31 of development, the corners of the egg cases naturally open, allowing water to enter the case and replace the vitelline fluid and the chemical gene inhibitor. Once the first egg case had opened, others were artificially opened to ensure that the treatment period remained constant between replicates. Egg cases remained sealed for 25 days before opening and were then allowed to develop for a further 35 days before fixation and morphological examination. After observing inhibition of denticle development (Fig. 9), a second round of drug treatments was conducted to examine the genetic effect of FGF inhibition via SU5402 treatment. WM*IS*H was undertaken to examine caudal denticle morphogenesis for SU5402-treated samples and compared to control samples (treated with DMSO). The concentration of SU5402 was increased, with 10 specimens receiving a 50-µl injection of a 5 mM stock solution of SU5402 in 1% DMSO in PBS, once every 24 h for 96 h, with each individual injection resulting in a ~50 µM concentration. 5 control samples were treated with one 50 µl injection of 1% DMSO in PBS, every 24 h for 96 h. Embryos were immediately fixed after the treatment period, before dissection and WM*IS*H took place. Two SU5402-treated tails and one DMSO control tail were used to investigate expression of each gene. The concentrations used for chemical treatments were gleaned from studies undertaking similar gene perturbation experiments in teleosts and chondrichthyans, and honed using preliminary drug treatment trials in *S. canicula* [37, 84, 85] (see Additional file 1).

### Whole mount in situ hybridisation (WMISH)

Digoxigenin-labelled (DIG) antisense riboprobes were designed using partial skate (*Leucoraja erinacea*) and catshark (*Scyliorhinus canicula*) EST assemblies [99] (SkateBase; skatebase.org) and the Vertebrate TimeCapsule (VTcap; transcriptome.cdb.riken.go.jp/vtcap). Riboprobes were cloned from *S. canicula* cDNA, and DIG-labelled antisense riboprobes were generated using the Riboprobe System Sp6/T7 kit (Promega). WM*IS*H was carried out in accordance with Fraser et al. [84]. Samples were rehydrated through a graded series of MeOH and PBS, and treated with proteinase K (1 µl/mg ProK for 60 min), to facilitate probe penetration. Next, samples were refixed in 4% PFA in PBS and incubated in pre-hybridisation buffer for 1 h at 61 °C. For the hybridisation stage, samples were placed in a shaker incubator overnight at 61 °C in 2 ml tubes (Eppendorf) containing 1 ml aliquots of hybridisation buffer and DIG-labelled antisense RNA probe. Samples were then washed in saline sodium citrate with 0.1% Tween-20 (SSCT), before incubation in blocking reagent (Roche). Antibody labelling occurred overnight at 4 °C in Maleic Acid Buffer with Tween-20 (MABT), using anti-DIG-ALP (0.2 µl/ml) (Roche). This was followed by a series of washes and 48-h incubation in MABT at 4 °C. For the colour reaction, BM purple (Roche) was applied at room temperature, until the staining was sufficiently strong to represent gene expression. For WM*IS*H undertaken upon SU5402 treated specimens, the colour reaction was run for the same length of time for SU5402-treated animals and DMSO controls. Samples were stored and imaged in 10% EtOH in PBS using Nikon SMZ1500 stereomicroscope. After WM*IS*H and imaging, embryos were post-fixed with 4% PFA and embedded in chick albumin cross-fixed with 2.5% glutaraldehyde. A Leica Microsystems VT1000 vibratome was used to cut sections at 30 μm. Vibratome sections were then mounted with Fluoromount (Sigma-Aldrich) and imaged using a BX51 Olympus Microscope.

### Section in situ hybridisation (SISH)

Fixed, dehydrated specimens were processed through a graded series of MeOH, chloroform and hot wax before being embedded in paraffin, and sectioned at 14 µm with a microtome (Leica RM2145). Sections were rehydrated from MeOH, and S*IS*H was carried out with solutions as described for WM*IS*H. Sections were incubated in pre-hybridisation buffer, before overnight incubation with a DIG-labelled antisense RNA probe. Sections were then run through post-hybridisation washes. Antibody labelling occurred overnight incubation with anti-DIG-AP (Roche). After post-antibody washes, BM purple (Roche) was used for the colour reaction. Sections were counterstained with haematoxylin and imaged using an Olympus BX51 Microscope and Olympus DP71 Universal digital camera attachment.

### Immunofluorescence

Sections were rehydrated from MeOH or EtOH as previously described for S*IS*H. Antigen retrieval occurred in hot 0.01 M sodium citrate (pH 6.0) for 10 min, before blocking and antibody labelling. Primary antibody labelling was undertaken using mouse anti-PCNA antibody (ab29; Abcam), overnight at 4 °C. Secondary antibody incubation was undertaken with goat anti-mouse AlexaFluor-488 (Thermo Fisher), before counterstaining with DAPI (Sigma-Aldrich). Slides were mounted with Fluoromount (Sigma-Aldrich). Imaging was undertaken with an Olympus BX61 upright epifluorescent Microscope and Olympus DP71 Universal digital camera attachment, and visualised with the software Volocity 6.3.

## Additional file

**Additional file 1.** Caudal denticle abortion from preliminary SU5402 treatment trial. A preliminary drug trial involved treating *S. canicula* embryos with a single injection resulting in a 50 µM concentration of SU5402 in the egg case, which then remained sealed for 30 days. The egg case then opened, and the embryo was allowed an additional 30-day recovery period before fixation. The sample was then stained with alcian blue and alizarin red. There is a clear vestige (B, black arrowhead), indicating a single denticle from the sequence was aborted, as a result of this SU5402 treatment.
